# Placental Vascular Resistance and Offspring Growth From Birth to Age 2 Years

**DOI:** 10.1001/jamanetworkopen.2025.43365

**Published:** 2025-11-12

**Authors:** Luli Xu, Jin’e Zhang, Kai Chen, Xiaohan Dong, Xiya Qin, Mingzhao Huang, DeMing Kong, Xiaoxuan Fan, Xiaofeng Mu, Lianting Hu, Yuji Wang, Zhiguo Xia, Jun Li, Aifen Zhou, Chao Xiong

**Affiliations:** 1 Wuhan Children’s Hospital (Wuhan Maternal and Child Healthcare Hospital), Tongji Medical College, Huazhong University of Science and Technology, Wuhan, Hubei, China; 2 Clinical Medical Research Center for Birth Defect Prevention and Treatment in Wuhan, Wuhan, China

## Abstract

**Importance:**

Umbilical artery Doppler indices provide indirect measures of placental function, with elevated vascular resistance associated with adverse perinatal outcomes, but their association with postnatal growth remains understudied.

**Objective:**

To examine the association between placental vascular resistance and early-childhood physical growth.

**Design, Setting, and Participants:**

This cohort study used hospital electronic medical records and Maternal and Child Health Information System data of all singleton live births from May 18, 2012, to October 30, 2023, at Wuhan Children’s Hospital (Wuhan Maternal and Child Health Hospital).

**Exposure:**

Placental vascular resistance was assessed through routine prenatal Doppler ultrasonography measurements of pulsatility index, resistance index, and systolic-to-diastolic ratio across 4 gestational windows (21-24 weeks, 25-28 weeks, 29-32 weeks, and 33-36 weeks).

**Main Outcomes and Measures:**

Child growth parameters from birth to age 2 years, including stunting, underweight, wasting, were defined as length for age, weight for age, and weight for length below the third percentile, respectively. Group-based multitrajectory modeling was conducted to characterize multiple trajectories of weight-for-age and length-for-age *z* scores.

**Results:**

A total of 52 660 mother-infant pairs were included (mean [SD] maternal age, 30.2 [3.9] years; 53.0% male offspring; mean [SD] gestational age, 38.8 [1.4] weeks). The prevalence rates of underweight, stunting, and wasting among children were 0.2% (n = 76), 0.3% (n = 101), and 0.8% (n = 284), respectively. Elevated placental vascular resistance at 21 to 24 weeks was associated with increased risks of childhood underweight (resistance index: odds ratio [OR], 1.27 [95% CI, 1.11-1.47]) and stunting (pulsatility index: OR, 1.29 [95% CI, 1.10-1.51]; resistance index: OR, 1.19 [95% CI, 1.05-1.35]; systolic-to-diastolic ratio: OR, 1.52 [95% CI, 1.11-2.07]). In the multitrajectory analysis, increased placental vascular resistance across all 4 gestational windows was significantly associated with a higher odds of children belonging to the persistently low, early-low and catch-up, and below-average groups and a lower odds of belonging to the persistently high group compared with the average group.

**Conclusions and Relevance:**

In this large cohort study of mother-child pairs followed up from pregnancy to childhood, elevated placental vascular resistance was associated with an increased risk of childhood underweight and stunting, highlighting the clinical importance of routinely monitoring Doppler indices during prenatal checkups.

## Key Points

**Question:**

What is the association of placental vascular resistance with children’s physical growth after birth?

**Findings:**

This cohort study in 52 660 mother-infant pairs found that elevated placental vascular resistance was associated with higher risks of underweight and impaired growth during early childhood.

**Meaning:**

These findings suggest the importance of routinely monitoring Doppler indices during prenatal checkups while remaining vigilant regarding the risk of impaired growth in children with elevated placental vascular resistance.

## Teaser

This cohort study examines the association between placental vascular resistance and early-childhood physical growth among children in China.

## Introduction

Key factors in the developmental origins of health and disease hypothesis include infants with low birthweight and who are small for gestational age (SGA).^1^ These adverse outcomes often result from developmental adaptations triggered by suboptimal fetal nutrition during this crucial period, leading to reduced fetal growth rates, smaller body size at birth, and altered tissue and organ development. Though not always apparent at birth, these changes can cause physiological issues later in life,^2^ contributing to conditions such as stunting (ie, low height for age) or wasting (ie, low weight for length). The substantial disease burden associated with growth impairment during early childhood highlights the critical need for early detection and intervention.^3,4^ Guided by the developmental origins of health and disease framework, we can investigate key influencing factors during early life stages to better understand and address these challenges.

The placenta plays a central role in mediating fetal nutrient and oxygen supply. Suboptimal placental growth or function that fails to meet fetal demands can trigger developmental adaptations with long-term consequences for postnatal growth.^5^ As placental disorders often originate from abnormalities in maternal placental circulation,^6^ placental vascular function serves as a more precise and dynamic indicator of placental function compared with gross placental weight. This function can be noninvasively evaluated throughout gestation using Doppler ultrasonography of the umbilical arteries, with key indices including the pulsatility index (PI), resistance index (RI), and systolic-to-diastolic ratio (S/D), which collectively reflect fetoplacental vascular resistance and fetal circulatory status. Elevated resistance may result from impaired placentation or suboptimal fetal vascular development.^7^

Previous studies have associated increased placental vascular resistance with smaller birth size and adverse birth outcomes such as preterm and SGA infants.^8^ However, few studies have examined the association between placental vascular resistance and childhood growth, often relying on single–time point measurements of length and weight.^5^ Early childhood is critical for physical growth, especially in preterm or SGA infants who often show catch-up growth.^9^ Longitudinal growth trajectories thus offer more comprehensive insight than single-point measurements. While conventional methods model weight and length trajectories separately to assess early growth,^10,11^ group-based multitrajectory modeling integrates both measures simultaneously, capturing their synergy and offering a more holistic view of early childhood development.^12^

This study explored the association between placental vascular resistance at different gestational periods and children’s physical growth from birth to age 2 years. We also investigated whether elevated placental vascular resistance during pregnancy is associated with early childhood growth impairment. The aim is to provide scientific evidence to enhance maternal and child health interventions.

## Methods

This hospital-based cohort study included all singleton live births at Wuhan Children’s Hospital (Wuhan Maternal and Child Health Hospital) from May 18, 2012, to October 30, 2023. The research protocol was approved by the Medical Ethics Committee of Wuhan Children’s Hospital. All participants provided written informed consent. The study followed the Strengthening the Reporting of Observational Studies in Epidemiology (STROBE) reporting guideline.^13^

### Study Population and Data Collection

We systematically collected maternal demographic characteristics, obstetric history, antenatal examinations, and delivery information through the hospital’s electronic medical record system. Placental function was assessed via Doppler ultrasonography measurements during routine prenatal visits. Child growth parameters were obtained from the Wuhan Maternal and Child Health Information Electronic System, which were routinely collected at 8 standardized time points (at birth and at 1, 3, 6, 8, 12, 18, and 24 months) by trained community health physicians.

The inclusion criteria were (1) singleton live birth without congenital anomalies, (2) complete maternal and infant records without logical inconsistencies in key variables (eg, last menstrual period, delivery date), (3) at least 1 Doppler ultrasonography examination between 21 and 36 weeks’ gestation, and (4) 3 or more complete anthropometric measurements from birth to age 2 years. A flowchart showing the selection of eligible mother-child pairs is presented in the eFigure in Supplement 1. The characteristics of missing data and participants lost to follow-up are presented in eTables 1 and 2 in Supplement 1.

### Measurement of Placental Vascular Resistance

Placental vascular resistance was assessed using umbilical artery blood flow velocity waveforms, with key measurements including peak systolic velocity, end-diastolic velocity, and time-averaged velocity across the cardiac cycle. The PI was calculated as the difference between peak systolic and end-diastolic velocities divided by the time-averaged velocity, while the RI was determined by dividing the same velocity difference by the peak systolic velocity. The S/D was computed as the peak systolic velocity divided by the end-diastolic velocity.^14^ Elevated PI, RI, and S/D values indicated increased placental vascular resistance, reflecting potential impairments in placental perfusion and fetal circulation.

Considering that Doppler ultrasonography is an integral part of routine prenatal care, we selected examination reports from 4 gestational windows (21-24 weeks, 25-28 weeks, 29-32 week, and 33-36 weeks) for analysis following the “Chinese Guideline for Preconception and Prenatal Care (2018).”^15^ When multiple examinations were available within the same window, the latest recorded measurement was included.

### Child Physical Measurement and Evaluation

The child’s length and weight measurements were routinely collected as part of standard child health care practice by trained community pediatricians at 8 scheduled follow-up visits (at birth and at 1, 3, 6, 8, 12, 18, and 24 months), with all data promptly recorded in Wuhan’s Maternal and Child Health Information Electronic System for dynamic growth monitoring. Weight-for-age *z* score (WAZ), length-for-age *z* score (LAZ), and weight-for-length *z* score (WFLZ) were calculated using World Health Organization child growth standards.^16^ Stunting, underweight, and wasting were defined as LAZ, WAZ, and WFLZ below the third percentile (LAZ < −1.88, WAZ < −1.88, WFLZ < −1.88).^17,18^ According to the World Health Organization’s recommendations for age adjustment, *z* scores for preterm infants were corrected for gestational age in analyses conducted at the 2-year time point.

### Covariate Assessment

Maternal basic information, including age, prepregnancy height, weight, and education level, as well as obstetric history (parity) and delivery details (date, mode, and infant sex), were obtained from the hospital’s electronic medical record system and the Wuhan Maternal and Child Health Information Electronic System. Prepregnancy body mass index (BMI) was calculated using self-reported weight in kilograms and measured height in meters squared from the first prenatal visit. Gestational age was determined based on the interval between the delivery date and the mother’s last menstrual period and confirmed by ultrasonography. Maternal education was categorized as college or above and less than college. Feeding patterns, collected during routine newborn follow-up visits, were classified into exclusive breastfeeding for the first 6 months or not.

### Statistical Analysis

The missing rates for prepregnancy BMI, education level, and feeding patterns were 10.1%, 1.1%, and 2.3%, respectively. Other variables, including maternal age, gestational week, infant sex, cesarean delivery, and parity were complete. Missing values were estimated using multiple imputations before analysis. All covariates were incorporated into the multiple imputation models, and 5 imputed datasets were generated prior to combining the results. Continuous variables are expressed as means and SDs, and categorical variables as frequencies with percentages.

Group-based trajectory modeling was used to identify subgroups of childhood growth trajectories as measured by WFLZ. Furthermore, group-based multitrajectory modeling was applied to identify subgroups of children with similar trajectories in WAZ and LAZ. This approach allows for capturing the correlation and potential synergistic effect of WAZ and LAZ. The optimal models were determined based on the following criteria: (1) improvement in the bayesian information criterion, (2) each trajectory group containing more than 5% of participants, and (3) high average posterior probabilities of group membership (>0.7).^19^ Subject matter knowledge also guided the decision on the optimal number of groups.

We used general linear models to examine longitudinal associations between placental vascular resistance across 4 gestational windows and offspring anthropometric indicators, including birth weight, birth length, WAZ, LAZ, and WFLZ at age 2 years, with adjustment for maternal age, educational attainment, prepregnancy BMI, parity, infant sex, and gestational age. For analyses involving WAZ, LAZ, and WFLZ at age 2 years, additional adjustments were made for the corresponding WAZ, LAZ, and WFLZ at birth, as well as for delivery mode and infant feeding patterns within the first 6 months of life. For binary growth outcomes (stunting, underweight, and wasting at age 2 years), Firth penalized logistic regressions were used to account for potential case-control imbalances.^20^ To reduce the false discovery rate, multiple corrections were applied to the results of the 3 models for each gestational window and each offspring anthropometric indicator. Multinomial logistic regressions were performed to assess the association of placental vascular resistance with both WFLZ trajectories and multiple trajectories of WAZ and LAZ. The odds ratio (OR) and its corresponding 95% CI were used to assess the effect size associated with a 1-SD increase in the independent variable.

Analyses were performed using R, version 4.4.1 (R Foundation for Statistical Computing) and SAS, version 9.4 (SAS Institute Inc), with trajectory analysis conducted using the PROC TRAJ macro. The significance level was set at a 2-sided *P* < .05.

## Results

### Baseline Characteristics of the Study Population

A total of 52 660 mother-infant pairs were included (Table 1). The mean (SD) maternal age at delivery was 30.2 (3.9) years, and the mean (SD) prepregnancy BMI was 21.2 (2.7). Among the newborns, 47.0% were female and 53.0% were male. The mean (SD) gestational age was 38.8 (1.4) weeks. The PI, RI, and S/D values exhibited an overall decreasing trend from the second trimester to the third trimester (detailed distributions presented in eTable 3 in Supplement 1).

**Table 1.** Participant Characteristics (N = 52 660)

Characteristic	Participants, No. (%)
Mothers	
Age, mean (SD), y	30.2 (3.9)
Prepregnancy BMI, mean (SD)	21.2 (2.7)
Maternal educational level	
Less than college	28 813 (54.7)
College or above	23 847 (45.3)
Cesarean delivery	
Yes	27 067 (51.4)
No	25 593 (48.6)
Parity	
Primiparous	36 731 (69.8)
Multiparous	15 929 (30.3)
Infants	
Sex	
Female	24 769 (47.0)
Male	27 891 (53.0)
Feeding patterns for 6 mo	
Mixed or artificial feeding	21 669 (41.2)
Breast feeding	30 991 (58.9)
Age, mean (SD), gestational wk	38.8 (1.4)
Assessment of placental vascular resistance, mean (SD)	
21-24 wk (n = 17 595)	
PI	1.03 (0.17)
RI	0.64 (0.07)
S/D	2.88 (0.46)
25-28 wk (n = 15 278)	
PI	0.89 (0.16)
RI	0.59 (0.07)
S/D	2.49 (0.38)
29-32 wk (n = 35 714)	
PI	0.84 (0.14)
RI	0.57 (0.08)
S/D	2.36 (0.33)
33-36 wk (n = 35 097)	
PI	0.77 (0.14)
RI	0.53 (0.07)
S/D	2.18 (0.31)

Abbreviations: BMI, body mass index (calculated as weight in kilograms divided by height in meters squared); PI, pulsatility index; RI, resistance index; S/D, systolic-to-diastolic ratio.

### Association Between Placental Vascular Resistance and Physical Growth

The associations between placental vascular resistance and child anthropometric indicators at birth and age 2 years are presented in eTables 4 and 5 in Supplement 1, respectively. At age 2 years, the prevalence of adverse growth outcomes among children was as follows: 76 (0.2%) were underweight, 101 (0.3%) experienced stunting, and 284 (0.8%) experienced wasting. The associations between placental hemodynamic parameters and these adverse growth outcomes are presented in Table 2. Elevated placental vascular resistance at gestational age 21 to 24 weeks was associated with increased risks of childhood underweight (RI: OR, 1.27 [95% CI, 1.11-1.47]) and stunting (PI: OR, 1.29 [95% CI, 1.10-1.51]; RI: OR, 1.19 [95% CI, 1.05-1.35]; S/D: OR, 1.52 [95% CI, 1.11-2.07]).

**Table 2.** Association Between Placental Vascular Resistance and Adverse Growth Outcomes at Age 2 Years^a^

Placental hemodynamic parameter	Underweight^b^			Stunting^c^			Wasting^d^		
	OR (95% CI)	*P* value	FDR	OR (95% CI)	*P* value	FDR	OR (95% CI)	*P* value	FDR
**21-24 wk (n = 10 371)**									
PI	1.13 (0.84-1.54)	.42	0.62	1.29 (1.10-1.51)	.001	0.003	1.16 (0.98-1.36)	.08	0.17
RI	1.27 (1.11-1.47)	.001	0.003	1.19 (1.05-1.35)	.006	0.009	1.11 (0.98-1.27)	.11	0.17
S/D	1.09 (0.77-1.56)	.62	0.62	1.52 (1.11-2.07)	.009	0.009	1.12 (0.92-1.37)	.27	0.27
**25-28 wk (n = 9729)**									
PI	0.68 (0.46-0.99)	.04	0.12	1.10 (0.79-1.52)	.58	0.68	0.97 (0.76-1.23)	.79	0.94
RI	0.75 (0.53-1.08)	.12	0.12	1.22 (1.04-1.44)	.02	0.06	0.99 (0.78-1.25)	.94	0.94
S/D	0.70 (0.48-1.04)	.08	0.12	1.07 (0.78-1.47)	.68	0.68	0.96 (0.77-1.21)	.76	0.94
**29-32 wk (n = 24 000)**									
PI	1.03 (0.80-1.32)	.81	0.94	1.19 (1.01-1.40)	.04	0.06	0.99 (0.87-1.14)	.93	0.93
RI	0.99 (0.73-1.34)	.94	0.94	1.19 (1.01-1.39)	.03	0.06	0.96 (0.81-1.13)	.60	0.93
S/D	1.02 (0.79-1.32)	.86	0.94	1.12 (0.89-1.41)	.35	0.35	0.98 (0.85-1.12)	.74	0.93
**33-36 wk (n = 23 064)**									
PI	0.89 (0.69-1.13)	.34	0.35	1.17 (0.99-1.39)	.07	0.21	0.95 (0.83-1.10)	.51	0.69
RI	0.89 (0.69-1.14)	.35	0.35	1.16 (0.92-1.46)	.20	0.27	0.97 (0.84-1.12)	.69	0.69
S/D	0.86 (0.67-1.11)	.24	0.35	1.12 (0.91-1.39)	.27	0.27	0.95 (0.83-1.10)	.51	0.69

Abbreviations: FDR, false discovery rate; OR, odds ratio; PI, pulsatility index; RI, resistance index; S/D, systolic-to-diastolic ratio.

^a^ The analysis involved fitting separate multivariable Firth penalized logistic regression models for each gestational window and for each independent variable (PI, RI, and S/D). The OR and its corresponding 95% CI were used to assess the effect size associated with a 1-SD increase in the independent variable.

^b^ The models were adjusted for maternal age, educational attainment, prepregnancy body mass index, parity, infant sex, gestational age, delivery mode (cesarean delivery), weight-for-age *z* score at birth, and infant feeding patterns within age 6 months. At age 2 years, 76 of 35 700 children (0.2%) were underweight.

^c^ The models were adjusted for maternal age, educational attainment, prepregnancy body mass index, parity, infant sex, gestational age, delivery mode (cesarean delivery), length-for-age *z* score at birth, and infant feeding patterns within age 6 months. At age 2 years, 101 of 35 700 children (0.3%) were underweight.

^d^ The models were adjusted for maternal age, educational attainment, prepregnancy body mass index, parity, infant sex, gestational age, delivery mode (cesarean delivery), weight-for-length *z* score at birth, and infant feeding patterns within age 6 months. At age 2 years, 284 of 35 700 children (0.8%) were underweight.

### Association Between Placental Vascular Resistance and Children’s Growth Trajectory Patterns

Figure, A illustrates 5 distinct WFLZ trajectory groups from birth to age 2 years identified among all the children. The persistently low group (5930 children [11.3%]) showed the lowest growth velocity with stable patterns. The early-low and catch-up group (6531 children [12.4%]) had the lowest WFLZ at birth but showed rapid acceleration until 12 months, achieving significant catch-up growth. The average group (18 962 children [36.0%]) exhibited median WFLZ at birth with stable trajectories, while the below-average group (18 461 children [35.1%]) followed parallel, but lower patterns. The persistently high group (2776 children [5.3%]) maintained the highest WFLZ throughout the observation with pronounced peak velocity.

**Figure.**
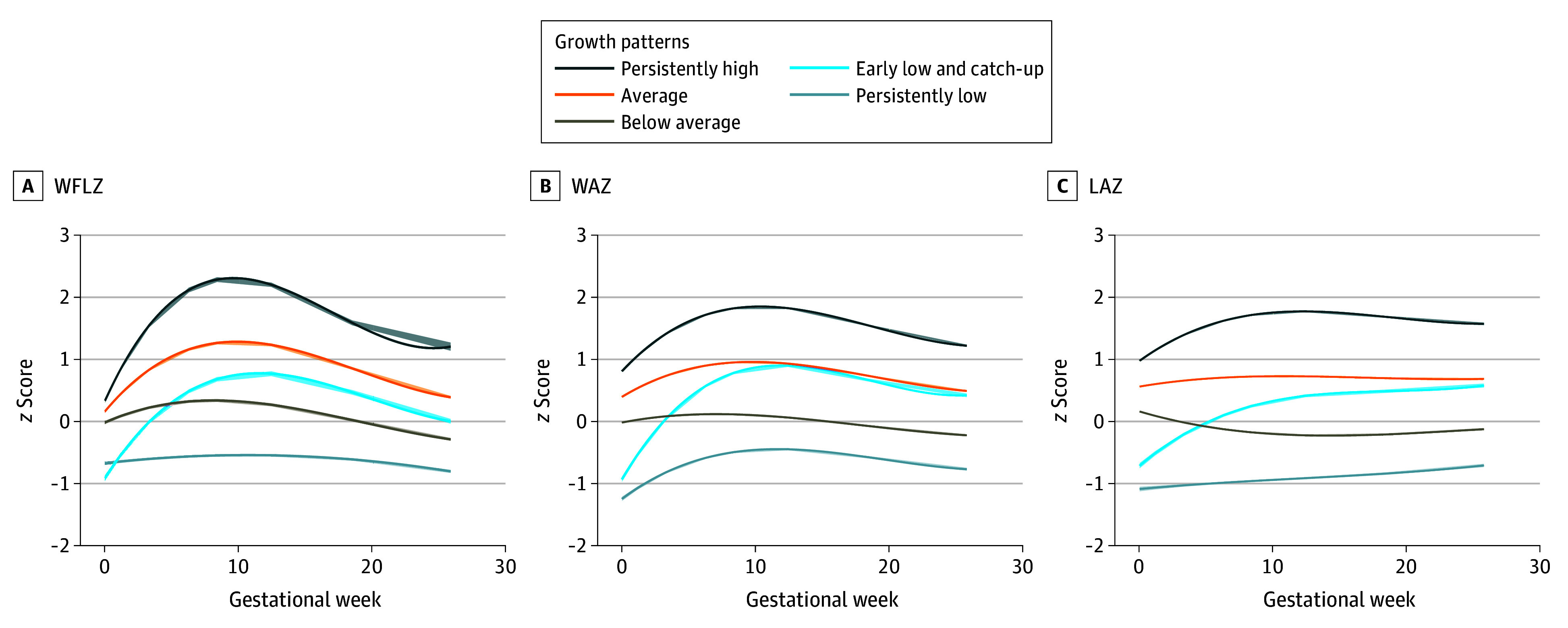
Weight-for-Length *z* Score (WFLZ), Weight-for-Age *z* Score (WAZ), and Length-for-Age *z* Score (LAZ) Trajectories The 5 trajectories of WFLZ were identified from group-based trajectory modeling, and the 5 WAZ and LAZ trajectories were identified from group-based multitrajectory modeling. The solid lines indicate point estimations, and the shading indicates 95% CI estimations.

Table 3 presents the associations between placental vascular resistance and WFLZ trajectories. Compared with the average group, the odds of being in the early-low and catch-up group increased with higher placental vascular resistance during the third trimester. Specifically, elevated PI, RI, and S/D at 33 to 36 gestational weeks were associated with an increased odds of being in the persistently low group (OR, 1.07 [95% CI, 1.04-1.11], 1.06 [95% CI, 1.02-1.10], and 1.07 [95% CI, 1.03-1.11], respectively).

**Table 3.** Associations Between Placental Vascular Resistance and Weight-for-Length Trajectories Within Age 2 Years^a^

Placental hemodynamic parameter	Persistently low group		Early-low and catch-up group		Below-average group		Persistently high group	
	OR (95% CI)	*P* value	OR (95% CI)	*P* value	OR (95% CI)	*P* value	OR (95% CI)	*P* value
**21-24 wk (n = 17 595)**								
PI	1.00 (0.95-1.05)	.98	1.04 (0.99-1.09)	.11	0.99 (0.96-1.03)	.69	1.00 (0.93-1.07)	.96
RI	1.00 (0.94-1.05)	.90	1.03 (0.98-1.09)	.19	1.00 (0.96-1.04)	.87	1.01 (0.93-1.08)	.89
S/D	1.00 (0.95-1.05)	.93	1.05 (1.00-1.10)	.08	1.00 (0.96-1.03)	.88	1.00 (0.94-1.07)	.95
**25-28 wk (n = 15 278)**								
PI	1.04 (0.99-1.10)	.14	1.03 (0.98-1.09)	.27	1.02 (0.98-1.06)	.42	1.03 (0.95-1.11)	.48
RI	1.04 (0.98-1.09)	.22	1.05 (1.00-1.11)	.07	1.04 (1.00-1.08)	.07	1.02 (0.94-1.10)	.68
S/D	1.06 (1.00-1.12)	.06	1.07 (1.01-1.13)	.02	1.04 (1.00-1.08)	.03	1.03 (0.95-1.12)	.45
**29-32 wk (n = 35 714)**								
PI	1.00 (0.97-1.04)	.86	1.07 (1.04-1.11)	<.001	1.01 (0.98-1.03)	.46	0.98 (0.94-1.03)	.51
RI	0.99 (0.95-1.03)	.68	1.03 (1.00-1.06)	.08	1.00 (0.98-1.03)	.91	1.03 (0.98-1.07)	.30
S/D	0.99 (0.95-1.02)	.42	1.06 (1.02-1.10)	.002	1.00 (0.97-1.02)	.87	0.99 (0.95-1.04)	.80
**33-36 wk (n = 35 097)**								
PI	1.07 (1.04-1.11)	<.001	1.08 (1.04-1.12)	<.001	1.02 (0.99-1.04)	.13	0.98 (0.94-1.03)	.52
RI	1.06 (1.02-1.10)	<.001	1.08 (1.04-1.11)	<.001	1.02 (1.00-1.05)	.09	0.99 (0.94-1.04)	.60
S/D	1.07 (1.03-1.11)	<.001	1.10 (1.06-1.14)	<.001	1.02 (0.99-1.05)	.15	0.99 (0.94-1.04)	.69

Abbreviations: OR, odds ratio; PI, pulsatility index; RI, resistance index; S/D, systolic-to-diastolic ratio.

^a^ Among the 52 660 participants, 5 distinct childhood growth trajectories were identified using group-based trajectory modeling: persistently low (n = 5930), early low and catch-up (n = 6531), below average (n = 18 461), average (n = 18 962) (reference group), and persistently high (n = 2776). The analysis involved fitting separate multivariable multinomial logistic regression models for each gestational window and for each independent variable (PI, RI, and S/D). The models were adjusted for maternal age, educational attainment, prepregnancy body mass index, parity, infant sex, gestational age, delivery mode (cesarean delivery), and infant feeding patterns within age 6 months. The OR and its corresponding 95% CI were used to assess the effect size associated with a 1-SD increase in the independent variable.

Multivariable trajectory analysis showed that a 5-group solution optimally characterized early growth patterns. Figure, B and C present the multitrajectory profiles for WAZ and LAZ, respectively. The groups were classified as persistently low (4200 children [8.0%]), early low and catch-up (4913 children [9.3%]), below average (15 892 children [30.2%]), average (20 257 children [38.5%]), and persistently high (7398 children [14.1%]) based on population distribution and growth characteristics.

Table 4 displays the associations between placental vascular resistance and multivariable trajectory groups for WAZ and LAZ. Compared with the average group, increased placental vascular resistance across all 4 gestational windows was significantly associated with an increased odds of being in the persistently low group, early-low and catch-up group, and below-average group. Conversely, increased placental vascular resistance was associated with a decreased odds of being in the persistently high group.

**Table 4.** Associations Between Placental Vascular Resistance and Multitrajectory Groups for Weight and Height Within Age 2 Years^a^

Placental hemodynamic parameter	Persistently low group		Early-low and catch-up group		Below-average group		Persistently high group	
	OR (95% CI)	*P* value	OR (95% CI)	*P* value	OR (95% CI)	*P* value	OR (95% CI)	*P* value
**21-24 wk (n = 17 595)**								
PI	1.13 (1.07-1.21)	<.001	1.15 (1.09-1.21)	<.001	1.05 (1.02-1.09)	.005	0.95 (0.91-1.00)	.047
RI	1.17 (1.10-1.24)	<.001	1.11 (1.05-1.18)	<.001	1.07 (1.02-1.11)	.002	0.95 (0.90-1.00)	.045
S/D	1.18 (1.11-1.25)	<.001	1.14 (1.07-1.20)	<.001	1.07 (1.03-1.11)	<.001	0.96 (0.91-1.00)	.07
**25-28 wk (n = 15 278)**								
PI	1.22 (1.14-1.30)	<.001	1.19 (1.12-1.27)	<.001	1.10 (1.06-1.15)	<.001	0.96 (0.91-1.01)	.11
RI	1.18 (1.10-1.26)	<.001	1.18 (1.11-1.26)	<.001	1.10 (1.05-1.14)	<.001	0.93 (0.88-0.98)	.004
S/D	1.22 (1.14-1.30)	<.001	1.19 (1.11-1.27)	<.001	1.11 (1.06-1.15)	<.001	0.92 (0.87-0.97)	.001
**29-32 wk (n = 35 714)**								
PI	1.14 (1.10-1.19)	<.001	1.09 (1.05-1.13)	<.001	1.04 (1.02-1.07)	.002	0.93 (0.90-0.96)	<.001
RI	1.10 (1.06-1.14)	<.001	1.06 (1.02-1.11)	.003	1.04 (1.01-1.07)	.005	0.92 (0.89-0.96)	<.001
S/D	1.14 (1.09-1.19)	<.001	1.10 (1.06-1.15)	<.001	1.04 (1.02-1.07)	.001	0.93 (0.90-0.97)	<.001
**33-36 wk (n = 35 097)**								
PI	1.26 (1.21-1.31)	<.001	1.20 (1.15-1.24)	<.001	1.04 (1.02-1.07)	.001	0.90 (0.87-0.93)	<.001
RI	1.17 (1.13-1.22)	<.001	1.15 (1.10-1.19)	<.001	1.03 (1.00-1.05)	.06	0.90 (0.87-0.93)	<.001
S/D	1.25 (1.19-1.30)	<.001	1.18 (1.14-1.23)	<.001	1.04 (1.02-1.07)	.002	0.90 (0.87-0.93)	<.001

Abbreviations: OR, odds ratio; PI, pulsatility index; RI, resistance index; S/D, systolic-to-diastolic ratio.

^a^ Among the 52 660 participants, 5 distinct childhood growth trajectories were identified using group-based multitrajectory modeling: persistently low (n = 4200), early low and catch-up (n = 4913), below average (n = 15 892), average (n = 20 257) (reference group), and persistently high (n = 7398). The analysis involved fitting separate multivariable multinomial logistic regression models for each gestational window and for each independent variable (PI, RI, and S/D). The models were adjusted for maternal age, educational attainment, prepregnancy body mass index, parity, infant sex, gestational age, delivery mode (cesarean delivery), and infant feeding patterns within age 6 months. The OR and its corresponding 95% CI were used to assess the effect size associated with a 1-SD increase in the independent variable.

## Discussion

This cohort study found that elevated placental vascular resistance not only is associated with smaller birth size but also maintains a persistent association with child growth trajectories through age 2 years. Specifically, it is significantly associated with an increased odds of children belonging to the persistently low and the early-low and catch-up growth groups. Notably, elevated placental vascular resistance was also associated with higher risks of underweight and stunting during early childhood.

Our findings align with growing evidence linking placental vascular resistance to fetal and child growth outcomes.^8,21^ Gaillard et al,^5^ using data from the Generation R Study, showed that elevated umbilical artery vascular resistance is associated with reduced fetal length and weight gain in the third trimester, leading to smaller birth size. These associations with poorer anthropometric measures (reduced stature and lower weight) persisted into childhood, consistent with our results. In our study, we have extended these observations by examining multiple growth parameters at age 2 years, including WAZ, LAZ, and WFLZ, along with risks of underweight, stunting, and wasting. Furthermore, we conducted stratified analyses across 4 clinically relevant gestational windows (defined by routine prenatal visit schedules) to identify critical periods of developmental susceptibility, thereby informing more targeted prenatal care strategies. Significant associations were observed between placental vascular resistance across all 4 gestational windows and birth length and weight. After adjusting for birth length and weight, the associations between placental vascular resistance across nearly all 4 gestational windows and body length and weight at age 2 years remained significant. Moreover, these measurements were associated with childhood underweight and stunting, particularly during the earlier gestational window of 21 to 24 weeks. This finding suggests that the long-term association between placental vascular resistance and child growth is not solely explained by fetal size and birth size. Therefore, a comprehensive evaluation integrating both fetal size and placental vascular resistance may provide a more holistic assessment of fetal health and development.

The early-life period represents a critical window for child development, characterized by marked growth dynamics that warrant particular attention.^10^ Longitudinal anthropometric changes during this period show more pronounced associations with later-life disease risk compared with single-time measurements.^22^ Notably, infants exhibiting restricted fetal growth followed by accelerated postnatal catch-up growth have shown elevated risks of developing insulin resistance, metabolic syndrome, obesity, and cardiovascular disease in adulthood.^23^ The Generation R Study found that elevated umbilical artery resistance in late pregnancy was associated with lower child height and weight up to age 6 years, as well as with adverse cardiometabolic indicators, such as higher BMI, fat mass, and blood pressure.^5^ Our findings that showed significant associations between placental vascular resistance and the early-low and catch-up growth trajectory suggest that these outcomes may be partly mediated through compensatory growth patterns in infants with restricted birth size. These results indicate that children with increased placental vascular resistance and small birth size who experience catch-up growth may experience long-term cardiometabolic risks. However, further research is needed to confirm these associations.

This study evaluated the trajectories of WAZ and LAZ, which are widely used indicators of child growth.^10,11,24,25^ While WAZ reflects short-term fluctuations in weight, LAZ captures long-term growth patterns, which are particularly relevant for understanding irreversible developmental outcomes, such as stunting.^26^ Unlike previous research, we used group-based multitrajectory modeling to identify subgroups with shared WAZ and LAZ patterns, a method validated for differentiating clinically significant subgroups of multiple fetal growth indicators.^12,27^ Our analysis revealed that while catch-up growth was observed for both parameters, linear growth trajectories (LAZ) exhibited less pronounced growth spurts compared with weight trajectories (WAZ), particularly among infants with low birth length. This finding aligns with previous literature on separate WAZ and LAZ trajectories.^24,25,26^ The differential growth patterns suggest that prenatal adverse factors may have a more severe and long-lasting association with height than weight gain, which deserves attention.

Current evidence has shown that Doppler multivessel assessment, a noninvasive prenatal examination, provides valuable information on fetal hypoxia and placental insufficiency, crucial for managing high-risk pregnancies.^28^ Our study further showed that Doppler-derived placental vascular resistance is associated with childhood growth stunting. Placental vascular resistance is influenced by maternal factors (eg, blood pressure, iron status, thyroid function)^29,30,31^ and environmental exposures (eg, air pollutants, bisphenols, phthalate metabolites).^32,33^ Furthermore, Barjaktarovic et al^29^ showed that placental hemodynamic alterations partially mediate the association between maternal thyroid function and birth weight. Lin et al^7^ also showed that increased umbilical artery impedance may mediate the effect of particulate matter (with a diameter of ≤2.5 µm) oxidative potential exposure on low fetal weight. These findings highlight the importance of monitoring and timely intervention to optimize placental circulation, potentially mitigating the adverse effects of prenatal exposures on offspring growth and development. From a public health perspective and based on the principles of tertiary prevention, it is first recommended that pregnant women take protective measures during pregnancy, especially in the early stages, to reduce exposure to harmful factors such as tobacco,^34^ air pollution,^32^ and organic pollutants,^33^ as these factors could increase placental vascular resistance. If placental vascular resistance becomes elevated, clinical interventions such as positional changes and oxygen therapy may improve placental blood circulation and alleviate placental hypoperfusion.^35,36^ Finally, for children who experienced high placental vascular resistance during pregnancy, closer attention should be paid after birth, including enhanced monitoring and nutritional supplementation, to reduce the risk of stunting in childhood.

### Limitations

This study had several limitations. First, the single-center nature of this study may limit the generalizability of the findings. Second, as the data were derived from routine clinical practice rather than a prospectively designed research cohort, the availability and completeness of data were limited, including missingness (particularly in early gestation) and the potential for selection bias. Third, despite adjusting for many demographic and clinical covariates, residual confounding was possible due to routine care data limitations. Fourth, the study focused only on umbilical artery resistance, and future research should include additional vascular assessments, such as uterine and middle cerebral arteries, for a more comprehensive evaluation.

## Conclusions

In this large cohort study of mother-infant pairs followed up from pregnancy to childhood, significant associations were observed between placental vascular resistance and anthropometric measures from birth to age 2 years. Notably, elevated placental vascular resistance was associated with a higher risk of stunting in early childhood. These results emphasize the clinical importance of early identification of high-risk fetuses through enhanced monitoring of placental vascular resistance during pregnancy while remaining vigilant about the risk of childhood growth impairment, particularly stunting.

## Notes

Supplement 1. **eFigure.** Flowchart of the Study Population **eTable 1.** Descriptive Characteristics of Participants With Doppler Ultrasonography and Without Doppler Ultrasonography **eTable 2.** Descriptive Characteristics of Participants With at Least 3 Follow-Ups and Less Than 3 Follow-Ups **eTable 3.** The Distribution of Placental Vascular Resistance **eTable 4.** Associations Between Placental Hemodynamics and Neonatal Size at Birth **eTable 5.** Associations Between Placental Hemodynamics and Anthropometric Indicators at Age 2 Years

Supplement 2.
Data Sharing Statement
