# Supplementary material for: Placental Vascular Resistance and Offspring Growth From Birth to Age 2 Years
Source: JAMA Netw Open. 2025 Nov 12;8(11):e2543365. doi: 10.1001/jamanetworkopen.2025.43365 (PMC12612932; doi:10.1001/jamanetworkopen.2025.43365)
Supplement: Supplement 1. — eFigure. Flowchart of the Study Population eTable 1. Descriptive Characteristics of Participants With Doppler Ultrasonography and Without Doppler Ultrasonography eTable 2. Descriptive Characteristics of Participants With at Least 3 Follow-Ups and Less Than 3 Follow-Ups eTable 3. The Distribution of Placental Vascular Resistance eTable 4. Associations Between Placental Hemodynamics and Neonatal Size at Birth eTable 5. Associations Between Placental Hemodynamics and Anthropometric Indicators at Age 2 Years [file jamanetwopen-e2543365-s001.pdf]

Supplemental Online Content

Xu L, Zhang J, Chen K, et al. Placental vascular resistance and offspring growth from birth to age 2 years. *JAMA Netw Open*. 2025;8(11):e2543365. doi:10.1001/jamanetworkopen.2025.43365

eFigure. Flowchart of the Study Population

eTable 1. Descriptive Characteristics of Participants With Doppler Ultrasound and Without Doppler Ultrasound

eTable 2. Descriptive Characteristics of Participants With at Least 3 Follow-Ups and Less Than 3 Follow-Ups

eTable 3. The Distribution of Placental Vascular Resistance

eTable 4. Associations Between Placental Hemodynamics and Neonatal Size at Birth

eTable 5. Associations Between Placental Hemodynamics and Anthropometric Indicators at Age 2 Years

This supplemental material has been provided by the authors to give readers additional information about their work.

© 2025 Xu L et al. *JAMA Network Open*.

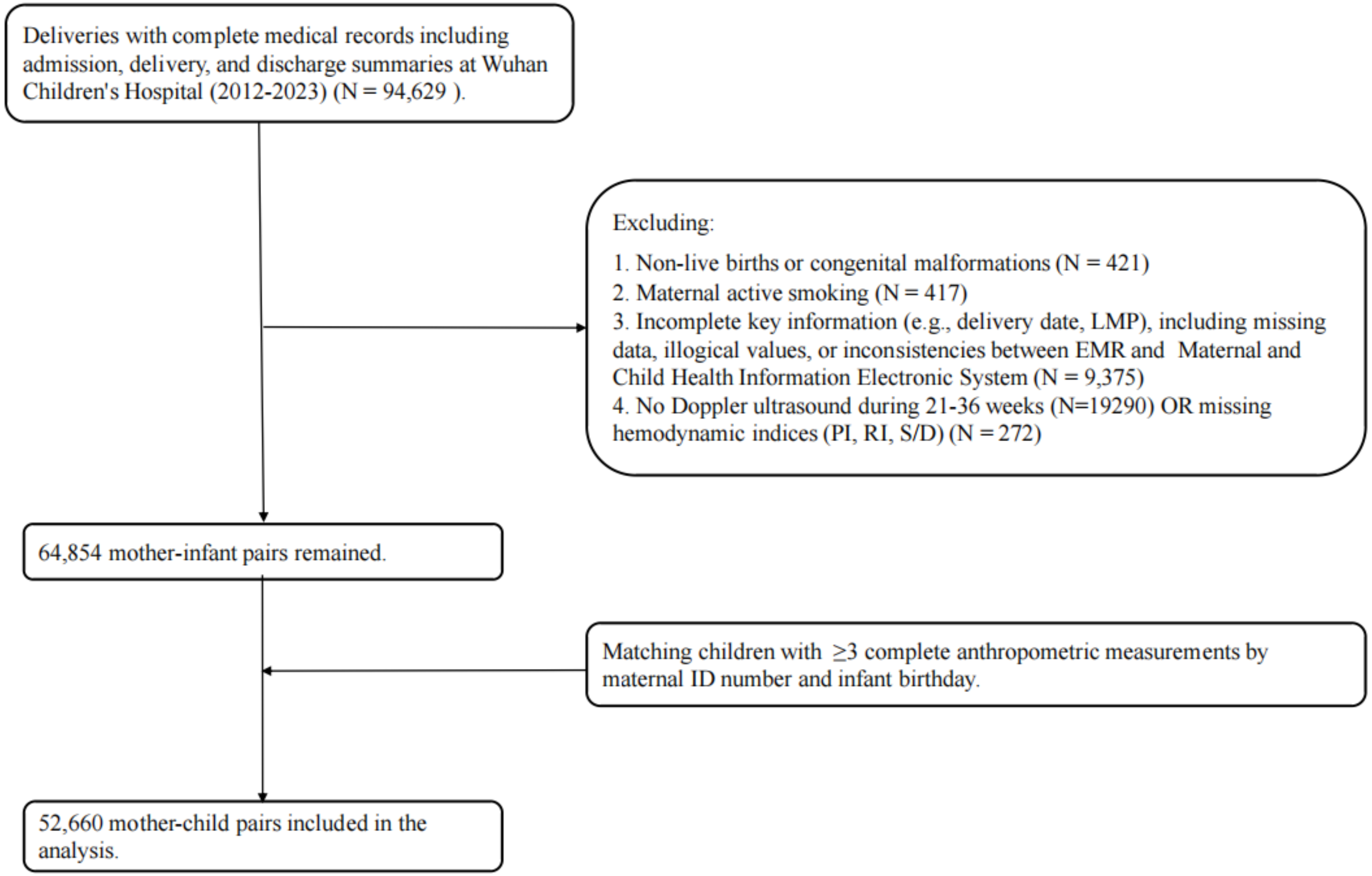

eFigure. Flowchart of the Study Population

© 2025 Xu L et al. *JAMA Network Open*.

eTable 1. Descriptive Characteristics of Participants With Doppler Ultrasound and Without Doppler Ultrasound

Characteristics	Participants with Doppler ultrasound (N=64854)	Participants without Doppler ultrasound (N=19562)	P value
**Mothers**			
Maternal age (years)	30.14 ± 3.90	29.75 ± 3.97	<0.001
Pre-pregnancy BMI (kg/m^2)	21.23 ± 2.77	21.03 ± 2.59	<0.001
Maternal educational level, n (%)			<0.001
Below college	35626 (55.94)	11193 (58.71)	
College or above	28055 (44.06)	7871 (41.29)	
Cesarean section, n (%)			<0.001
Yes	33471 (51.61)	11442 (58.49)	
No	31383 (48.39)	8120 (41.51)	
Parity, n (%)			0.11
Primiparous	45353 (69.93)	13797 (70.53)	
Multiparous	19501 (30.07)	5765 (29.47)	
**Newborns**			
Infant sex, n (%)			<0.001
Male	34459 (53.13)	10116 (51.71)	
Female	30395 (46.87)	9446 (48.29)	
Gestational week (weeks)	38.67 ± 1.55	39.08 ± 1.12	<0.001

© 2025 Xu L et al. *JAMA Network Open*.

eTable 2. Descriptive Characteristics of Participants With at Least 3 Follow-Ups and Less Than 3 Follow-Ups

Characteristics	Participants with at least three follow-ups (N=52660)	Participants with less than three follow-ups (N=12194)	P value
**Mothers**			
Maternal age (years)	30.22 ± 3.89	29.81 ± 3.88	<0.001
Pre-pregnancy BMI (kg/m^2)	21.24 ± 2.77	21.16 ± 2.79	0.006
Maternal educational level, n (%)			<0.001
Below college	28760 (55.24)	6866 (59.10)	
College or above	23304 (44.76)	4751 (40.90)	
Cesarean section, n (%)			0.03
Yes	27067 (51.40)	6404 (52.52)	
No	25593 (48.60)	5790 (47.48)	
Parity, n (%)			0.04
Primiparous	36731 (69.75)	8622 (70.71)	
Multiparous	15929 (30.25)	3572 (29.29)	
**Newborns**			
Infant sex, n (%)			0.07
Male	27891 (52.96)	6568 (53.86)	
Female	24769 (47.04)	5626 (46.14)	
Gestational week (weeks)	38.75 ± 1.40	38.33 ± 2.04	<0.001
**Placental hemodynamic assessment**			
21-24 weeks			
PI	1.03 ± 0.17	1.03 ± 0.17	0.86
RI	0.64 ± 0.07	0.64 ± 0.07	0.71
S/D	2.88 ± 0.46	2.88 ± 0.46	0.76

© 2025 Xu L et al. *JAMA Network Open*.

25-28 weeks			
PI	0.89 ± 0.16	0.89 ± 0.16	0.22
RI	0.59 ± 0.07	0.59 ± 0.09	0.1
S/D	2.49 ± 0.38	2.50 ± 0.39	0.19
29-32 weeks			
PI	0.84 ± 0.14	0.84 ± 0.14	0.31
RI	0.57 ± 0.08	0.57 ± 0.07	0.27
S/D	2.36 ± 0.33	2.37 ± 0.33	0.02
33-36 weeks			
PI	0.77 ± 0.14	0.78 ± 0.14	<0.001
RI	0.53 ± 0.07	0.54 ± 0.08	<0.001
S/D	2.18 ± 0.31	2.19 ± 0.31	0.001

Data are presented as mean ±SD or n (%). Pulsatility index (PI), resistance index (RI), systolic/diastolic ratio (S/D)

© 2025 Xu L et al. *JAMA Network Open*.

eTable 3. The Distribution of Placental Vascular Resistance

	Mean ± SD	10th	25th	50th	75th	90th
21-24 weeks (N=17595)						
PI	1.03 ± 0.17	0.82	0.92	1.03	1.14	1.23
RI	0.64 ± 0.07	0.56	0.61	0.65	0.69	0.71
S/D	2.88 ± 0.46	2.30	2.55	2.86	3.20	3.50
25-28 weeks (N=15278)						
PI	0.89 ± 0.16	0.70	0.79	0.89	0.99	1.07
RI	0.59 ± 0.07	0.50	0.55	0.60	0.64	0.66
S/D	2.49 ± 0.38	2.00	2.22	2.49	2.74	2.95
29-32 weeks (N=35714)						
PI	0.84 ± 0.14	0.66	0.75	0.84	0.94	1.01
RI	0.57 ± 0.08	0.48	0.53	0.58	0.61	0.64
S/D	2.36 ± 0.33	1.93	2.12	2.37	2.60	2.80
33-36 weeks (N=35097)						
PI	0.77 ± 0.14	0.59	0.67	0.77	0.87	0.95
RI	0.53 ± 0.07	0.44	0.49	0.54	0.58	0.61
S/D	2.18 ± 0.31	1.80	1.96	2.18	2.40	2.60

Pulsatility index (PI), resistance index (RI), systolic/diastolic ratio (S/D)

© 2025 Xu L et al. *JAMA Network Open*.

eTable 4. Associations Between Placental Hemodynamics and Neonatal Size at Birth[a]

	Birth weight			Birth length		
	β (95%CI)	*P*	FDR	β (95%CI)	*P*	FDR
21-24 weeks (N=17588)						
PI	-0.02 (-0.02, -0.01)	<0.001	<0.001	-0.06 (-0.08, -0.04)	<0.001	<0.001
RI	-0.02 (-0.02, -0.01)	<0.001	<0.001	-0.06 (-0.08, -0.04)	<0.001	<0.001
S/D	-0.02 (-0.02, -0.01)	<0.001	<0.001	-0.07 (-0.09, -0.05)	<0.001	<0.001
25-28 weeks (N=15266)						
PI	-0.02 (-0.03, -0.02)	<0.001	<0.001	-0.08 (-0.10, -0.06)	<0.001	<0.001
RI	-0.02 (-0.03, -0.02)	<0.001	<0.001	-0.08 (-0.10, -0.06)	<0.001	<0.001
S/D	-0.03 (-0.03, -0.02)	<0.001	<0.001	-0.09 (-0.11, -0.07)	<0.001	<0.001
29-32 weeks (N=35697)						
PI	-0.02 (-0.03, -0.02)	<0.001	<0.001	-0.07 (-0.09, -0.06)	<0.001	<0.001
RI	-0.02 (-0.02, -0.01)	<0.001	<0.001	-0.06 (-0.07, -0.04)	<0.001	<0.001
S/D	-0.02 (-0.03, -0.02)	<0.001	<0.001	-0.08 (-0.09, -0.06)	<0.001	<0.001
33-36 weeks (N=35092)						
PI	-0.04 (-0.05, -0.04)	<0.001	<0.001	-0.14 (-0.15, -0.12)	<0.001	<0.001
RI	-0.03 (-0.04, -0.03)	<0.001	<0.001	-0.11 (-0.12, -0.10)	<0.001	<0.001
S/D	-0.04 (-0.05, -0.04)	<0.001	<0.001	-0.14 (-0.16, -0.13)	<0.001	<0.001

[a]The analysis involved fitting separate multivariate general linear models for each gestational window and for each independent variables: pulsatility index (PI), resistance index (RI), and systolic/diastolic ratio (S/D). The β and its corresponding 95% confidence interval (CI) are utilized to assess the effect size associated with a one-standard-deviation increase in the independent variable. The models were adjusted for maternal age, educational attainment pre-pregnancy BMI, parity, infant sex, and gestational age.

© 2025 Xu L et al. *JAMA Network Open*.

eTable 5. Associations Between Placental Hemodynamics and Anthropometric Indicators at Age 2 Years[a]

	WAZ[b]			LAZ[c]			WFLZ[d]		
	β (95%CI)	*P*	FDR	β (95%CI)	*P*	FDR	β (95%CI)	*P*	FDR
21-24 weeks (N=10371)									
PI	-0.01 (-0.02, 0.01)	0.32	0.32	-0.02 (-0.03, 0.00)	0.08	0.08	-0.01 (-0.02, 0.01)	0.48	0.48
RI	-0.02 (-0.03, 0.00)	0.01	0.03	-0.03 (-0.04, -0.01)	0.003	0.009	-0.01 (-0.03, 0.00)	0.13	0.315
S/D	-0.01 (-0.03, 0.00)	0.09	0.135	-0.02 (-0.04, 0.00)	0.02	0.03	-0.01 (-0.03, 0.01)	0.21	0.315
25-28 weeks (N=9729)									
PI	-0.02 (-0.03, 0.00)	0.04	0.04	-0.03 (-0.05, -0.02)	<0.001	<0.001	-0.01 (-0.03, 0.01)	0.4	0.4
RI	-0.02 (-0.03, 0.00)	0.02	0.03	-0.03 (-0.05, -0.01)	0.001	0.001	-0.01 (-0.03, 0.00)	0.16	0.36
S/D	-0.02 (-0.03, 0.00)	0.02	0.03	-0.04 (-0.05, -0.02)	<0.001	<0.001	-0.01 (-0.03, 0.01)	0.24	0.36
29-32 weeks (N=24000)									
PI	-0.01 (-0.02, 0.00)	0.005	0.015	-0.03 (-0.04, -0.01)	<0.001	<0.001	-0.01 (-0.02, 0.00)	0.1	0.3
RI	-0.01 (-0.02, 0.00)	0.03	0.03	-0.03 (-0.05, -0.02)	<0.001	<0.001	0.00 (-0.01, 0.01)	0.76	0.76
S/D	-0.01 (-0.02, 0.00)	0.01	0.015	-0.03 (-0.04, -0.02)	<0.001	<0.001	0.00 (-0.01, 0.01)	0.53	0.76
33-36 weeks (N=23064)									
PI	-0.01 (-0.02, 0.00)	0.21	0.26	-0.02 (-0.03, 0.00)	0.009	0.015	-0.01 (-0.02, 0.00)	0.08	0.12
RI	-0.01 (-0.02, 0.00)	0.18	0.26	-0.01 (-0.02, 0.00)	0.04	0.04	-0.01 (-0.02, 0.00)	0.05	0.12
S/D	-0.01 (-0.02, 0.00)	0.26	0.26	-0.01 (-0.03, 0.00)	0.01	0.015	-0.01 (-0.02, 0.00)	0.13	0.13

© 2025 Xu L et al. *JAMA Network Open*.

[a]The analysis involved fitting separate general linear models for each gestational window and for each independent variables: pulsatility index (PI), resistance index (RI), and systolic/diastolic ratio (S/D). The β and its corresponding 95% confidence interval (CI) are utilized to assess the effect size associated with a one-standard-deviation increase in the independent variable. The models were adjusted for maternal age, educational attainment pre-pregnancy BMI, parity, infant sex, gestational age, delivery mode (cesarean section) and infant feeding patterns within six months of age.

[b]The models were adjusted for maternal age, educational attainment pre-pregnancy BMI, parity, infant sex, gestational age, delivery mode (cesarean section), weight-for-age Z-score at birth and infant feeding patterns within six months of age.

[c]The models were adjusted for maternal age, educational attainment pre-pregnancy BMI, parity, infant sex, gestational age, delivery mode (cesarean section), length-for-age Z-score at birth and infant feeding patterns within six months of age.

[d]The models were adjusted for maternal age, educational attainment pre-pregnancy BMI, parity, infant sex, gestational age, delivery mode (cesarean section), weight-for length Z-score at birth and infant feeding patterns within six months of age.

Weight-for-age Z-score (WAZ), length-for-age Z-score (LAZ), weight-for length Z-score (WFLZ), pulsatility index (PI), resistance index (RI), systolic/diastolic ratio (S/D)

© 2025 Xu L et al. *JAMA Network Open*.
