# Supplementary material for: Placental Vascular Resistance and Offspring Growth From Birth to Age 2 Years
Source: JAMA Netw Open. 2025 Nov 12;8(11):e2543365. doi: 10.1001/jamanetworkopen.2025.43365 (PMC12612932; doi:10.1001/jamanetworkopen.2025.43365)
Supplement: Supplement 2. — Data Sharing Statement [file jamanetwopen-e2543365-s002.pdf]

Data Sharing Statement

Data
Data available: No

Additional Information
Explanation for why data not available: Because the data involve patient privacy, the datasets used and/or analyzed during the current study are available from the corresponding authors upon reasonable request.
